# Gut microbiota and cardiac arrhythmia

**DOI:** 10.3389/fcimb.2023.1147687

**Published:** 2023-04-25

**Authors:** Hongxuan Fan, Xuchang Liu, Zhaoyu Ren, Xiaoning Fei, Jing Luo, Xinyu Yang, Yaya Xue, Fenfang Zhang, Bin Liang

**Affiliations:** ^1^ Department of Cardiology, The Second Hospital of Shanxi Medical University, Taiyuan, Shanxi, China; ^2^ Department of Urology, The First Hospital of Shanxi Medical University, Taiyuan, Shanxi, China; ^3^ Clinical College, Shanxi Medical University, Taiyuan, Shanxi, China; ^4^ Department of Cardiology, Yangquan First People’s Hospital, Yangquan, Shanxi, China

**Keywords:** GM, metabolites, cardiac arrhythmia, mechanism, review

## Abstract

One of the most prevalent cardiac diseases is cardiac arrhythmia, however the underlying causes are not entirely understood. There is a lot of proof that gut microbiota (GM) and its metabolites have a significant impact on cardiovascular health. In recent decades, intricate impacts of GM on cardiac arrythmia have been identified as prospective approaches for its prevention, development, treatment, and prognosis. In this review, we discuss about how GM and its metabolites might impact cardiac arrhythmia through a variety of mechanisms. We proposed to explore the relationship between the metabolites produced by GM dysbiosis including short-chain fatty acids(SCFA), Indoxyl sulfate(IS), trimethylamine N-oxide(TMAO), lipopolysaccharides(LPS), phenylacetylglutamine(PAGln), bile acids(BA), and the currently recognized mechanisms of cardiac arrhythmias including structural remodeling, electrophysiological remodeling, abnormal nervous system regulation and other disease associated with cardiac arrythmia, detailing the processes involving immune regulation, inflammation, and different types of programmed cell death etc., which presents a key aspect of the microbial-host cross-talk. In addition, how GM and its metabolites differ and change in atrial arrhythmias and ventricular arrhythmias populations compared with healthy people are also summarized. Then we introduced potential therapeutic strategies including probiotics and prebiotics, fecal microbiota transplantation (FMT) and immunomodulator etc. In conclusion, the GM has a significant impact on cardiac arrhythmia through a variety of mechanisms, offering a wide range of possible treatment options. The discovery of therapeutic interventions that reduce the risk of cardiac arrhythmia by altering GM and metabolites is a real challenge that lies ahead.

## 1 Introduction

Non-communicable diseases are the leading cause of death globally (Cao et al., 2018). Cardiovascular diseases (CVD) are responsible for 46% of the mortality due to non-communicable diseases in worldwide (Mendis et al., 2015). Sudden fatal arrhythmias are responsible for a significant proportion of total mortality of the cardiovascular population (Osadchii, 2017). At the same time, arrhythmia as a risk factor for the onset of CV events such as myocardial infarction, stroke and other cardiovascular diseases seriously damages human life and health. Therefore, the understanding of pathogenesis, prevention, diagnosis and treatment of arrhythmia has become a problem to be solved urgently.

A related study suggest that arrhythmia associated with nutrient intake and metabolism (Nalliah et al., 2018). Notably, the adult gut system is a highly diverse and dynamic ecosystem of 39 trillion microbes (Tierney et al., 2019). They enter the human body through food, water and air at first and ultimately constitute the normal microbiota of the gut tract in humans, which exert specific physiological functions in gut mucosal protection, immune regulation, and maintenance of nutritional metabolism. Constantly updated evidence shows that the gut microbiota (GM) not only plays vital role in maintaining the gut normal nutrient metabolism of the host (Sonnenburg et al., 2016), but also is inseparable from the progress and development of diseases. For this reason, the relationship between GM disorder and arrhythmia is non-negligible.

**Figure 1:**
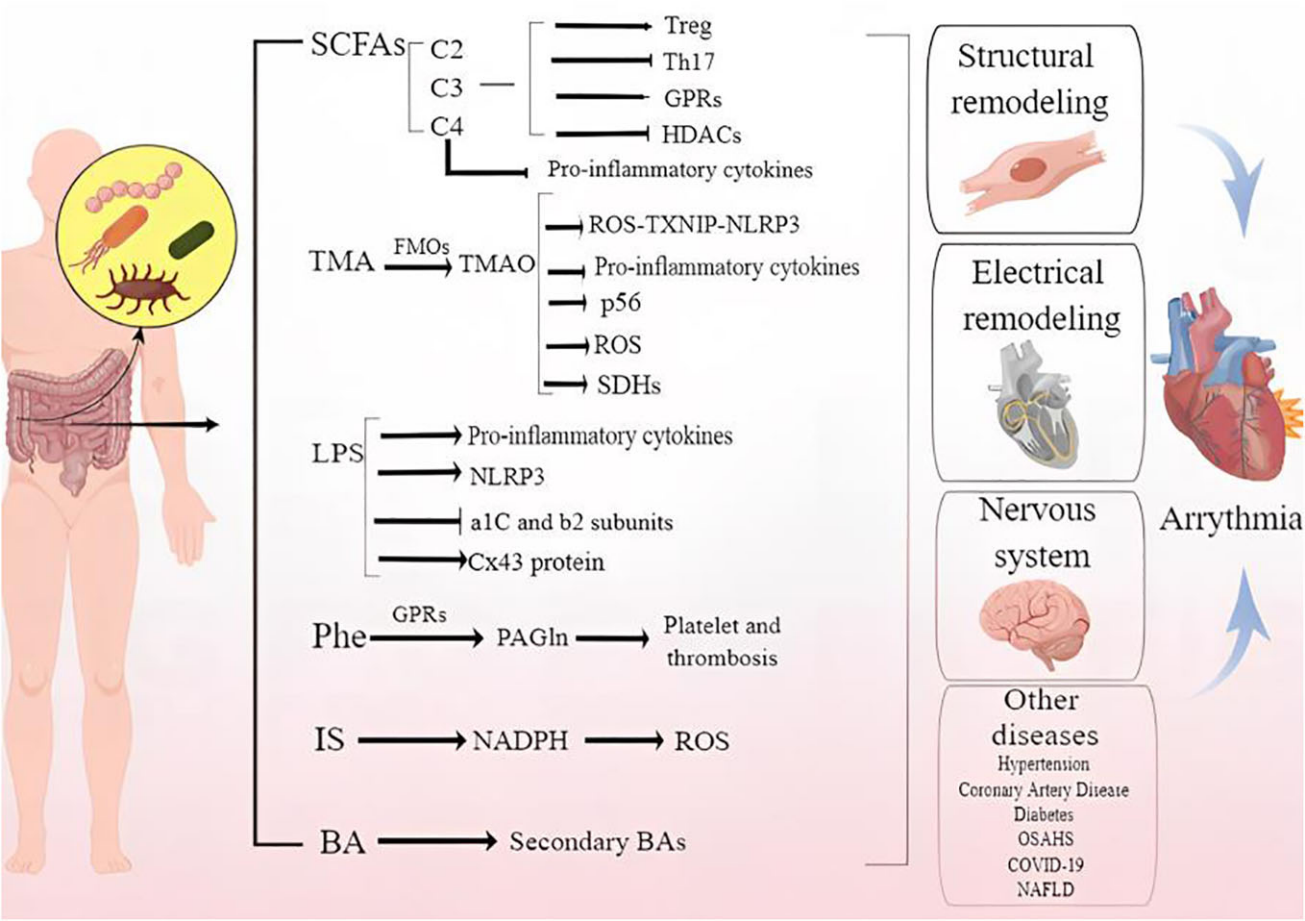
Demonstrates the mechanism by that gut microbiota and metabolites cause arrhythmia.

Several arrhythmogenic mechanisms is by now a widely studied, such as cardiac fibrosis, oxidative stress as well as changes in ion channel function, but there are still vacancies. In recent years, the exploration of the related mechanisms of dysbiosis of the GM, including *in situ* immunization disorders and the increase of harmful metabolites, is gradually being revealed. On this basis, a small observational study (Svingen et al., 2018) showed abnormal metabolites of gut dysbiosis linked to AF. However, the specific impact mechanism of GM imbalance on arrhythmia is not yet clear. In conclusion, we have carried out a comprehensive description and summary of this issue, hoping to provide theoretical basis and new treatment strategies for the prevention and treatment of arrhythmia. **Figure 1** demonstrates the potential mechanism by that gut microbiota and metabolites cause arrhythmia.

## 2 GM and dysbiosis

### 2.1 GM

Trillions of microorganisms reside in the body of a healthy adult (Sender et al., 2016). Notably, the GM is known as the “second largest genome” of the human body along with the human cell genome (Lynch and Pedersen, 2016). Therefore, researchers have focused most of their attention on GM. The GM of healthy adults is relatively stable. In the gut microenvironment, seven classes of bacteria thrive (*Firmicutes, Bacteroidetes, Actinobacteria, Fusobacteria, Proteobacteria, Verrucomicrobia*, and *Cyanobacteria*) *(* Adak and Khan, 2019), with *Phylum Bacteroides* and *Phylum Chlamydomonas* accounting for more than 90% of all gut microorganisms (Qin et al., 2010). Although GM has a relatively consistent function in healthy individuals (Human Microbiome Project Consortium, 2012), participating in normal physiological processes such as digestion, absorption, and excretion. GM plays an important role in maintaining the health homeostasis of the body (Tang et al., 2017).

Bacteria in the intestine can be divided into the following three categories according to their symbiotic relationship with the human body: i. The beneficial effects of probiotics (Suez et al., 2019),; ii. conditional pathogenic bacteria,; iii. pathogenic bacteria (Barko et al., 2018),, and requires reliance on accurate GM technology detection means to discern the state of GM balance. In recent years, macro-genomic combined with 16sRNA high-throughput sequencing technologies have been widely used to decipher the Alpha, Beta diversity and OTU abundance of microbial populations and thus explore the relationship between microbes and diseases.

### 2.2 Gut immunity and GM homeostasis

The gut has an independent immune system. gut mucosal immunity is a special existence. It is the body’s first barrier against the invasion of pathogens and plays an important role in microbial colonization in humans and in protecting the integrity of the host gut wall barrier.

The effect of GM on gut mucosal immunity is first reflected in promoting the development of the host immune system (Fiedler et al., 2013)..GM induces gut type 3 Innate lymphoid cells(ILCs) to produce Interleukin-17(IL-17), increasing plasma Granulocyte Colony-Stimulating Factor (G-CSF) levels and neutrophil numbers in a Toll-like receptor 4 (TLR4) and myeloid differentiation factor 88 (MyD88)-dependent manner,which is essential for combating E. coli sepsis in neonatal mice (Deshmukh et al., 2014). As the first line of defense against pathogens, macrophages play a role in tissue maintenance by initiating wound repair. During gut injury, through Repeat and Pyrin domain containing 3(NLRP3) inflammatory reaction, selective entero-gut bacterial symbiosis can reduce pre-IL-1 β Cut into mature IL-1 β, Stimulate goblet cells to secrete mucin to maintain the integrity of mucous membrane and induce the development of gut mucosa-associated lymphoid tissue through DC cells(DC) recognition of bacteria and their metabolites (Seo et al., 2015) (Furusawa et al., 2013). To create a balance between bacterial tolerance and immunity, DCs recognize gut bacteria through TLR and activate signaling pathways to release various cytokines (Maynard et al., 2012; Rooks and Garrett, 2016). ILC’s function by restricting macrophage production of the proinflammatory cytokines IL1b, IL12, IL23, IL22, and interferon-γ(IFN-γ), whereas gut macrophages play a key role in boosting mucosal Type 17 help T cells (Th17)responses (Gong et al., 2019). Host- immune interactions play a crucial role in the pathogenesis of inflammatory disease, including inflammatory bowel disease, Crohn’s disease, rheumatoid arthritis (Pickard et al., 2017).

### 2.3 Metabolites of GM

Carbohydrates, proteins, and fats are essential for the body’s physiological processes, but only partially digested. As a result of excess macronutrients temporarily stored in the intestine, microbial metabolism can produce biologically active metabolites that can affect the host’s physiological processes directly or indirectly once the nutrient intake exceeds the digestive rate (Nicolas and Chang, 2019). Pyruvate is formed by glycolyzing simple sugars from carbohydrates and dietary fiber. By further metabolizing these intermediate metabolites, short chain fatty acids (SCFAs) can be produced (Oliphant and Allen-Vercoe, 2019). A number of factors make SCFAs the most studied and extensively researched metabolic end products of GM. These include their ability to act as signaling molecules for G-protein coupled receptors (GPCRs), their ability to inhibit histone deacetylases, and their effect on host gene expression (Tan et al., 2014).. Compounds such as amines, phenols, indoles, and sulfur-containing compounds play a significant role in the brain-gut axis and are connected to conditions like depression, anxiety, Alzheimer’s, and uremia (Wang X, et al., 2019). Approximately 4-5 grams of fat per day reach the colon, which receives less than 5% of dietary fat. It is gut microbes that use lipases to break down triglycerides, choline, and phosphatidylcholine (lecithin) to create polar head groups (Schoeler et al., 2019).

#### 2.3.1 SCFAs

The GM metabolizes carbohydrates through a broadly diverse set of enzymes that are mostly absent in mammals and belong to the large family of carbohydrate-active enzymes (Garron and Henrissat, 2019) The GM breaks down dietary fiber to create organic acids, gases, and significant quantities of SCFAs. The three primary forms of SCFAs are acetate (C2), propionate (C3), and butyrate (C4) (Belzer et al., 2017). G protein-coupled receptors (GPCRs) of which GPR41 and GPR43 are found in a range of tissues, including adipose, enteroendocrine, and inflammatory cells, are capable of recognizing SCFAs. Mice’s protective immunological responses and tissue inflammation are mediated by chemokines and cytokines, which are produced by mitogen-activated protein kinase (MAPK) signaling when SCFAs activate GPR41 and GPR43 expression in gut epithelial cells (ECs) (Kim et al., 2013). Through the control of genes, SCFAs can have a variety of impacts on the host, such as changes in metabolism, cell differentiation, and proliferation. The expression of 5-20% of human genes can actually be regulated by butyric acid, according to a number of studies (Donohoe et al., 2012). Pro-inflammatory cytokines including IL-12 and TNF-α are thought to be inhibited by butyric acid (Artis, 2008).

#### 2.3.2 IS

A person’s gut bacteria produce uremic toxins, which are absorbed by the blood and eliminated by the kidneys. In the event of changes in microbial composition, excessive uremic toxins may be secreted, resulting in renal tubular cell damage. Protein-bound uremic toxins, indoxyl sulfate (IS) and p-cresol sulfate (PCS), are produced by colonic bacterial fermentation of dietary proteins (Gong et al., 2019). A link has been demonstrated between serum IS levels and the presence of aortic calcification, pulse wave velocity, the first episode of heart failure(HF), all-cause mortality, and cardiovascular mortality in chronic kidney disease (CKD) patients (Cao et al., 2015). When free (but not total) p-Cresol sulfate concentrations are greater than 0.051 mg/100 ml, there are strong associations between p-Cresol sulfate and cardiovascular mortality (Liabeuf et al., 2010). Indole-3-acetic acid binds to the AhR transcription factor, which regulates oxidative stress, vascular inflammation, and atherosclerosis (Gondouin et al., 2013).

#### 2.3.3 TMAO

TMAO is a metabolite produced by the host liver after being derived from the GM. As the precursor to TMAO, TMA requires GM to form. Choline, betaine, carnitine, and TMAO can be converted to TMA by the microbiota in the gut. In the liver, TMA enters *via* the portal circulation. It is then oxidized by flavin monooxygenases (FMOs) to generate TMAO. TMAO in the circulation can stimulate platelet hyperreactivity, cause foam cells to form, trigger inflammation, and reverse cholesterol transport. These effects can lead to the progression of atherosclerosis, heart failure, or chronic kidney disease (Zhang et al., 2021). TMAO levels were found to be associated with an increased risk of deaths and major adverse cardiovascular events in a study of 283 people (Obeid et al., 2016). A higher proportion of gut bacteria strains has been observed in HF patients, which is consistent with previous studies. This suggests that changes in GM may affect TMAO levels by regulating gut TMA synthesis (Romano et al., 2015). According to a prospective study of acute coronary patients, TMAO can accurately predict short- and long-term adverse cardiovascular events (Li X. S, et al., 2017).

#### 2.3.4 LPS

Circulating lipopolysaccharide (LPS) concentrations were found to be a reliable predictor of severe adverse cardiac events in a cohort of patients with AF in recent observational research (Hug et al., 2018). Inflammation, immunity, and vascular function are all regulated by increased levels of LPS and other bacterial wall products, which are most likely produced by the GM. When the gut barrier is breached, LPS from Gram-negative bacteria can enter the host circulation and are then largely detected by TLRs (Toll-like receptors) on the surface of immune cells (Li et al., 2016). TLR signaling triggers the release of pro-inflammatory cytokines in response to bacterial ligand binding, which controls the host’s pro-inflammatory state. Akkermansia muciniphila attenuates atherosclerotic lesions by ameliorating metabolic endotoxemia-induced inflammation through restoration of the gut barrier in apolipoprotein E-deficient (ApoE(-/-)) mice (Yoshida et al., 2018). Similarly, the administration of gram negative bacteroides (vulgatus and dorei) producing pentaacylated and tetraacylated lipid A by gavage to ApoE -/- mice will reduce colitis, endotoxemia and atherosclerosis (Pastori et al., 2017).

#### 2.3.5 PAGln

PAGln was linked to serious adverse cardiovascular events and CVD (myocardial infarction, stroke, or death). The essential amino acid phenylalanine (Phe) is a dietary precursor to the metabolite phenylacetylglutamine (PAGln), which is generated by GM. According to the study, the gut microbial porA gene promotes the conversion of Phe to phenylacetic acid in the human body. Thereafter, the host produces PAGln and phenylacetylglycine (PAGly). Through GPRs, PAGln triggers platelet activation and thrombosis (Nemet et al., 2020).

#### 2.3.6 Bile acids

The majority of total BAs, or primary BAs, are derived from cholesterol in the host liver. Following their discharge into the gut (duodenal) lumen, these primary BAs undergo changes that are dependent on the GM, leading to the emergence of other BA species. gut bacteria can further convert into secondary BAs such as ursodeoxycholic acid through dehydrogenation, dihydroxylation, or differential isomerization. Probiotic microbiota has the ability to change the BA ratio, resulting in lower levels of secondary BAs (eg., ursodeoxycholic acid (UDCA)) and higher levels of primary BAs (eg., chenodeoxycholic acid (CDCA)). Following systemic adsorption, several secondary BAs interact with various host nuclear receptors, including the farnesoid X receptor (FXR), liver X receptor(LXR), and pregnane X receptor(PXR), which in turn impacts host physiology (Kemis et al., 2019).

## 3 GM profile in patients with arrhythmia

Cardiac arrhythmias are the abnormalities or perturbations in the normal activation or beating of heart myocardium. There are many types of cardiac arrhythmias,among which atrial arrhythmia and ventricular arrhythmia have a high incidence and the most serious impact on patients.The range of cardiac rhythm disorder varies from harmless to life-threatening, from observation and waiting to emergency intervention and treatment (Donahue, 2017). Atrial fibrillation (AF) is the most common atrial arrhythmia, affecting more than 37 million people worldwide (Chugh et al., 2014). Malignant VAs, including persistent ventricular tachycardia (VT) and ventricular fibrillation (VF), are the main causes of cardiac death after myocardial infarction. For decades, researchers have been committed to exploring the etiology, mechanism and treatment of arrhythmia. At the same time, the constantly updated evidence shows that intestinal microflora is not only closely related to the maintenance of human health and homeostasis, but also closely related to the occurrence and development of various diseases, including cardiac arrhythmias (Peng et al., 2018).

So in this review, we discussed the mechanism of the relationship between intestinal microflora and arrhythmia.

### 3.1 GM profile in patients with atrial arrhythmia

Macrogenomics and metabolomics, as important research tools for studying changes in gut microbiome composition as well as their metabolites, respectively, play a major role in exploring the relationship between gut microbiome and cardiac arrythmia, providing new insights into our understanding of the role of gut microbiome changes and their metabolites in the development of cardiac arrythmia. Recently, it has been reported that there is a preliminary understanding of the relationship between gut microbiome and AF, and gut microbiome and its metabolites are closely related to the development of AF in terms of atrial structural remodeling and electrical remodeling. Gut microbiota genus and metabolites changes between AF and control group were summarized in **Tables 1**, **2**.

**Table 1:** Gut microbiota genus change between arrhythmia and control groups.

Article	Genomics	Study design	Increased genus	Decreased genus
(Zuo et al., 2019a) Zuo et al., 2019	Metagenomic sequencing	50 AF vs 50 control	*Eubacterium, Bifidobacterium, Roseburia, Ruminococcus, Blautia, Streptococcus, Dorea, Veillonella, Enterococcus, Coprobacillus*	*Faecalibacterium, Prevotella, Alistipes*, *Oscillibacter, Sutterella, Butyricicoccus*, *Bilophila, Flavonifractor, Hungatella, Anaerotruncus*
(Tabata et al., 2021)Tabata et al., 2021	16S rRNA sequencing	34 AF vs 66 control	*Parabacteroides, Lachnoclostridium*, *Streptococcus, Alistipes*	*Enterobacter*
(Zuo et al., 2020) Zuo et al., 2020	Metagenomic sequencing	50 AF vs 50 control	*Lactobacillus, Bifidobacterium*, *Roseburia,Ruminococcus, Blautia, Streptococcus*, *Dorea, Eubacterium*	*Prevotella, Oscillibacter*
		20 perAF vs 30 pAF	*Holospora, Methylovulum*	
(Zuo et al., 2019b)Zuo et al., 2019	Metagenomic sequencing	20 perAF vs 20 control	*Eubacterium, Blautia, Dorea*, *Butyricicoccus, Coprococcus, Lachnospira*, *Tyzzrella, Streptococcus*, *Anaerostipes, Paraprevotella*	*Butyricicoccus, Paraprevotella*
		8 pers>12m vs 12 pers< 12m	*Thermosinus, Anaeroarcus*	*Faecalibacterium, Corynebacterium*, *Mycobacterium*
(Li J. et al., 2020) Li et al., 2020	Metagenomic sequencing	40 RFCA AF vs 50 control	*Bifidobacterium, Blautia, Eubacterium*, *Ruminococcus, Streptococcus, Dorea*, *Coprococcus, Dialister*	*Prevotella, Oscillibacter*
		17 reAF vs 23 non-reAF	*Marinitoga, Rufibacter*, *Nitrosomonadaceae, Lentisphaeraceae*	
(Highet et al., 2014)Highet et al., 2014	16S rRNA sequencing	52 SIDS vs 102 control	*Cl. Difficile, Cl. Innocuum*, *B. thetaiotamicron, S. aureus*	

**Table 2:** Gut microbiota metabolites between AF and control groups.

Article	Metabolomics	Compared with the control group, metabolites in the stool and serum of the study group have the same change trend			Increased metabolites	Decreased metabolites
		General	Increased	Decreased		
(Zuo et al., 2019a) Zuo et al., 2019	Non-targeted LC/MS	16	3	13	Chenodeoxycholic acid LysoPC(15:0) Indole	LysoPE(0:0/20:0), Choline, Linoleic acid, Oleic acid, Cholic acid, α-Linolenic acid α-kamlolenic acid, L-Threonine, L-Lysine, L-Phenylalanine, 2-Phenylacetamide, L-Isoleucine, L-Methionine
(Zuo et al., 2020) Zuo et al., 2020	Non-targeted LC/MS	8	4	4	LysoPC(15:0) LysoPE(0:0/16:0) Chenodeoxycholic acid L-Tyrosine	LysoPE(0:0/20:0) α-Linolenic acid L-Threonine L-Isoleucine
(Zuo et al., 2019b) Zuo et al., 2019	Non-targeted LC/MS	10	4	6	Stearamide|, LysoPC(16:0) Bilirubin, Octadecanedioic acid	Oleic Acid, Corticosterone, L-Methionine, L-Isoleucine, L-Leucine, Choline
(Li J, et al., 2020) Li et al., 2020	Non-targeted LC/MS	8	4	4	LysoPC(15:0), LysoPE(0:0/16:0) Chenodeoxycholic acid, sebacic acid	LysoPE(0:0/20:0), Corticosterone α-Linolenic acid, uracil

Lately, a few studies have reported evidence in characterizing the gut microbiota shift in atrial fibrillation compared to healthy control. Zuo et al. (2019a) analyzed the composition of GM and its metabolites in 50 healthy controls versus 50 AF patients using macro-genomics and metabolomics techniques. It was found that AF patients had a significantly higher number of genes and increased intra-sample diversity (Shannon index) in their GM, representing a higher abundance and diversity of their gut flora. The higher number of genes and genus species suggest that there may be multiple harmful bacterial overgrowth in AF patients. Genera such as *Ruminococcus*, *Streptococcus*, *Veillonella* and *Enterococcus* are more enriched in AF patients, and *Ruminococcus* has pro-inflammatory properties that have been reported to be associated with the development of inflammatory bowel disease (Joossens et al., 2011), as well as studies that that administration of C. tumefaciens all the way into germ-free mice increased plasma levels of IFN-γ, IL-17, and IL-22; *Streptococcus* has also been shown to be elevated in populations with hypertension (Li J, et al., 2017),chronic heart failure (Cui et al., 2018) and acute cardiovascular events. Zuo et al. performed metabolomic analysis of stool and serum samples from healthy subjects and patients with AF, in which the abundance of BAs, OAs, linoleic acid and α-linolenic acid was significantly reduced. It has been shown that oleic, linoleic, and α-linolenic acids have been found to demonstrate cardioprotective effects, and linoleic and α-linolenic acids may prevent and terminate arrhythmias caused by lysophosphatidylcholine or acylcarnitine (Jiang et al., 2017). A research from Japan showed Enterobacter was depleted, while *Parabacteroides*, *Lachnoclostridium*, *Streptococcus*, and *Alistipes* were enriched in AF patients compared to control subjects (Tabata et al., 2021). It has been well-established that branched chain amino acids (BCAAs) are positively associated with a number of CVD events (heart failure, coronary artery disease and hypertension so on). The BCAA metabolic pathway is consistently downregulated in cardiac tissue of humans and animals with HF, resulting in a buildup of BCAAs and BCKAs, which may contribute to HF pathophysiology through various different pathways (McGarrah and White, 2023). Besides, a new study showed chronically elevated branched chain amino acid levels are pro-arrhythmic (Portero et al., 2022). Nevertheless, the role of BCAA in cardiovascular disease is controversial and seems to be time-dependent. It has been demonstrated that acute BCAA treatment has cardioprotective effects during cardiac ischemia-reperfusion and reduces infarct size (Satomi et al., 2020). Contrarily, it was discovered that prolonged BCAA administration increased myocardial sensitivity to ischaemia-reperfusion injury by boosting glycolysis and fatty acid oxidation, which in turn worsened cardiac dysfunction and remodeling after myocardial infarction (Li Y, et al., 2020). However, studies discussed in this review suggested that BCAAs were significantly reduced in AF population compared to the control group. We speculate this may be related to the younger age of the control group in several articles and the mechanisms underlying dysregulated BCAA metabolism and cardiac arrhythmias need to be further investigated. In conclusion, these articles are suggestive of a decrease in protective intestinal bacteria, a decrease in cardiovascular protective metabolites and an increase in metabolites hazardous to health that often accompany AF population compared to healthy population.

AF is a progressive disease. It can be classified into paroxysmal AF and persistent AF according to the duration of the attack. The same combined analysis approach was applied to explore the GM in different types of AF (Zuo et al., 2020). Fifty healthy people, 30 patients with pAF, and 20 patients with psAF were included in the study. Compared to controls, the abundance of *ruminal cocci* spp. and *streptococci* spp. etc. was increased in the pAF and psAF groups, and metabolomic analysis showed that metabolites such as CDCA were enriched in pAF and psAF patients. Combined analysis showed that enriched CDCA was positively correlated with enriched genera of C. tumefaciens and Streptococcus spp. in both pAF and psAF. CDCA was found to be positively correlated with the left atrial LVA, which can lead to atrial myocyte apoptosis (Wang et al., 2020). To identify the most specific and functionally important microbiota in the GM of pAF and psAF patients, a Spielman correlation analysis to explore the relationship between differential microbiota and atrial internal diameter parameters within the two groups. *Methylovulum* and *Holospora* were significantly more abundant in the psAF group than in the pAF group and were significantly associated with increased atrial internal diameter parameters. Zuo et al. (2019b) used combined macrogenomics and metabolomics analysis to study the relationship between the duration of persistent AF and GM. The study included 20 healthy individuals, 20 patients with persistent AF, 12 with a duration of AF less than 12 months (Pers < 12m) and 8 with a duration of AF greater than 12 months (Pers > 12m). The genus Vibrio butyric acid showed a decreasing trend with duration, *Vibrio butyric acid* spp. can produce short-chain fatty acids and have beneficial effects on the host, and *Vibrio faecalis* spp. showed an increasing trend with duration *Vibrio faecalis* spp. can greatly contribute to the development of coronary heart disease in patients with chronic schizophrenia (Nguyen et al., 2019). All of this evidence suggests that a decrease in beneficial bacteria and/or an increase in pathogenic bacteria may be associated with the pathological mechanisms of psAF. Besides, nine genera were significantly correlated with six of the fecal metabolites. These 6 metabolites were in turn associated with AF duration and CHA2DS2-VASc scores representing the severity of atrial remodeling in AF patients. Genera common to both groups, such as *Tyzzerella*, *anaerobic clubbed bacilli*, *dolichomyces*, and *true bacilli* belonging to the genus *Stearamide* were positively correlated, and *Stearamide* was also positively correlated with the CHA2DS2-VASc score, but these genera were negatively correlated with choline, which was negatively correlated with the CHA2DS2-VASc score. Notably the abundance of choline significantly decreased with increasing AF duration and was negatively correlated with *Enterobacter* spp. These interrelated microbial and metabolite alterations suggest that multiple microorganisms may be involved in the development of psAF through interactions with multiple host metabolites. These present studies provide a comprehensive description of the GM profiles of PAF and psAF patients and different duration types of persAF patients. This seems to indicate that some specific genera and metabolites may be closely linked to the duration of atrial fibrillation and may even play a critical role in heart structural remodeling.

Patients with AF are at risk of recurrence after catheter ablation. Maintenance of sinus rhythm after catheter ablation and avoidance of recurrence are difficult to predict and control. Li J, et al., (2020) studied stool samples from 50 healthy controls as well as 40 patients with AF who had undergone catheter ablation (23 non-recurrent, 17 recurrent, mean follow-up 15.6 months). Macrogenomics, total gene count, alpha-diversity, beta-diversity, PCA, and PCoA analysis revealed that both the non-RAF and recurrent atrial fibrillation(RAF) groups had significantly altered alpha and beta diversity compared to healthy controls. In RAF, increased gut microbial diversity and a deeper degree of dysbiosis predicted an overproliferation of harmful bacteria. Most of the 198 genera with significant differences between the non-RAF and RAF groups showed similar trends compared to the healthy controls. The genus Enterobacter was significantly lower, whereas *Parabacteroides*, *Lachnoclostridium*, *Streptococcus*, and *Alistipes* were significantly higher in AF patients than in control subjects (Tabata et al., 2021). In brief, reduced *Faecalibacterium* abundance in the intestine is thought to boost inflammatory cytokines, cause low-grade inflammation, and hence cause RAF. These related microbial and metabolic changes imply that the involved microorganisms may be able to influence the recurrence of AF by interacting with certain host metabolites.

### 3.2 GM profile in patients with ventricular arrhythmia

VA is one of the most common clinical arrhythmias. A previous study by Ghali (Ghali et al., 1991) found that left ventricular septal or posterior wall thickness was an important contributor to the increased incidence and complexity of VA. Fetal left ventricular growth may be one of the causes of VA. In recent years, the relationship between gut microbes and fetal growth and development has been studied extensively. Guzzardi et al. (2019) found that fetal left ventricular growth was associated with the composition of the gut microbiota at birth, in which the thickness of the neonatal left ventricular posterior wall (LVPW) was associated with a lower diversity of gut microbiota, depletion of bacterial (*Lactobacillales*, etc.) associated with anti-remodeling effects and enrichment of bacterial (*Enterobacteriales*, etc.) associated with inflammatory functions.

Gut microbiota-induced pro-inflammatory profiles promote ventricular growth during fetal development, and early microbiota-based modulation in pregnant women may reduce the incidence of VA and poor outcomes. Schuijt et al. believed that the intestine can affect critically ill individuals by encouraging systemic inflammation and infection. The host defense mechanism disintegrates in the presence of stress and mucosal hypoxia, resulting in the transfer of bacteria and toxins (Taylor, 1998; Alverdy and Chang, 2008), thus causing myocardial damage through endogenous and exogenous inflammatory responses (Sun et al., 2019). According to studies, ventricular tachyarrhythmias are directly associated to 50% to 75% of sudden cardiac deaths (SCD) (Tang et al., 2017). Recently, a higher proportion of *Cl. difficile*, *Cl. innocuum* and *B. thetaiotaomicron* was found in the intestine of SIDS infants compared to control healthy infants (Highet et al., 2014). *B. Thetaiotomicron* seems to mediate the formation of mucosal - gut barrier and help protect against pathogenic invasion through its influence on the expression of species-specific protein antibiotics (Xu and Gordon, 2003). Pathogenic Clostridium can damage EC tight junctions or trigger an inflammatory response, both of which can compromise the integrity of the gut wall (Sappington et al., 2002). Thus, early infant VA may be related to the established of an aberrant GM. For infants’ health and early development, it is thought to be crucial to create a healthy GM.

VA has been studied extensively, but the specific mechanisms by which intestinal flora and metabolites act are still unclear, and more research is needed.

## 4 GM dysbiosis and cardiac arrhythmia

In recent years, more and more relevant studies have proved that GM disturbance is associated with ischemic cardiomyopathy, heart failure, and even arrhythmia. Although the mechanism of cardiac arrhythmia is not fully understood, in recent decades, researchers have partially elucidated the pathological process of atrial fibrillation (AF). AF is primarily caused by ectopic pacemakers and susceptible atrial matrix (Hu et al., 2015). There are three main mechanisms of ventricular tachycardia (VT): increased automaticity, reentrant excitation, and trigger mechanisms (Lerman et al., 2014). It has been shown that the gut microbe-derived metabolite TMAO is associated with hyperactivity of the cardiac sympathetic nervous system (CSNS) and also plays an important role in the regulation of arrhythmias (Yu et al., 2017). The following will explain how GM uses metabolites, inflammatory factors, and immune cells as mediators to eventually lead to the occurrence of tachyarrhythmias through nerves, blood, and direct effects on myocardial tissue.

### 4.1 Structural remodeling

Myocardial remodeling refers to certain changes in the original electrophysiological and histological characteristics of the myocardium during the progression of tachyarrhythmias. One of the main manifestations of myocardial remodeling is structural remodeling, including myocardial cell apoptosis, atrial matrix fibrosis, etc (Begg et al., 2016). This is mainly due to various reasons such as hypertension, coronary heart disease, diabetes, obesity, etc., which lead to changes in the structure, shape and quantity of endoplasmic reticulum, mitochondria and other organelles in cardiomyocytes, resulting in cardiomyocyte hypertrophy, apoptosis, necrosis and myocardial interstitial fibrosis. Finally, the muscle wall is continuously thickened and the volume of the cardiac chamber is continuously expanded, which ultimately promotes the occurrence and maintenance of tachyarrhythmias.

#### 4.1.1 Immune response

The reduction of immune inflammation could attenuate cardiac hypertrophy, fibrosis, vascular dysfunction, and hypertension through SCFAs produced by gut bacteria. SCFAs are bacterial metabolites produced by colonic bacterial fermentation of indigestible resistant starch (RS) and non-starch polysaccharides (NSP), the main component of dietary fiber), mainly including acetate, propionic acid and butyric acid. SCFA stimulate colonic blood flow and absorption of fluids and electrolytes (Topping and Clifton, 2001). The anti-inflammatory effect on immune cells is realized by short-chain fatty acids, for example, the differentiation and inhibitory function of CD25+Foxp3+ Treg can be induced by propionic acid (Arpaia et al., 2013; Smith et al., 2013). T cell regulatory phenotype can be promoted by propionic acid under Th17 polarization conditions (Park et al., 2015). Myocardial hypertrophy, fibrosis, vascular dysfunction and hypertension could be attenuated by propionic acid. Cardioprotective effect of propionic acid through inhibition of histone deacetylases (HDACs) or through GPRs and olfactory receptors. At the same time, systemic immune inflammation manifested as a reduction in splenic effector memory T cell frequency and splenic Th17 could be attenuated by propionic acid. Meanwhile, the protection of the heart by propionic acid is mainly dependent on the action of regulatory T cells (Bartolomaeus et al., 2019). Short-chain fatty acids may prevent or delay the onset of arrhythmias by reducing the adverse cardiovascular effects mentioned above of the inflammatory response.

#### 4.1.2 Inflammation

Various substances produced by microorganisms in the digestive tract may cause arrhythmias through inflammatory pathways. Activated ROS-TXNIP-NLRP3 inflammasome by TMAO lead to inflammation and endothelial dysfunction of human umbilical vein (Sun et al., 2016). The MAPK and nuclear factor-κB signaling cascade (NF-κB) could be activated by TMAO injection and inflammatory markers such as cyclooxygenase 2, interleukin 6, E-selectin, and intercellular adhesion molecular-1 (ICAM-1). With significantly increased inflammatory markers, activated leukocytes were recruited to induce vascular inflammation, which suggests a likely contributory mechanism for TMAO-dependent enhancement in atherosclerosis and cardiovascular risks (Seldin et al., 2016). IS could induce pulmonary vein (PV) and atrial arrhythmogenesis through oxidative stress (Chen et al., 2015). Koike et al. (2019) found reduced serum IS levels in CKD patients with sinus rhythm who were AF with high IS after Radiofrequency Current Catheter Ablation (RFCA), suggesting that serum IS may not only induce AF, but may also be influenced by AF. Choline is a necessary component in human body and it is used for composing neurotransmitter acetylcholine, methyl donor betaine and phospholipid. Therefore, choline participates in the key physiological functions of all stages in the cell cycle. It is known that choline is the metabolite generated from lipid phosphatidylcholine by GM (Wang et al., 2011). It is worth mentioning that a previous study concluded that choline could inhibit activation of p38 MAPK mediated by ROS and regulate Ca2+-mediated calcineurin signal transduction pathway (Wang et al., 2012). All of the above demonstrate the vital role of choline in regulating inflammation. Significantly elevated LPS and glucose lead to upregulation of NOD-like receptor protein (NLRP)-3 inflammasome expression, which it also may indirectly contribute to the process in hosts with fibrotic atria, MCC950, a potent and selective inhibitor of NLRP3 inflammasomes, reduces atrial fibrosis and AF susceptibility (Zhang et al., 2022).

#### 4.1.3 Myocardial hypertrophy and myocardial fibrosis

Myocardial hypertrophy and myocardial fibrosis are the crucial causes of arrhythmia. GM can ferment pyruvate to produce succinic acid, lactic acid, acetyl coenzyme A, and further metabolize into short chain fatty acids (SCFAs), including C2, propionic acid, butyric acid and valeric acid. Some of these products are closely related to myocardial hypertrophy and myocardial fibrosis. It has been shown that propionic acid can reduce inflammatory reaction, and reduce myocardial hypertrophy, fibrosis, vascular dysfunction and hypertension in mice with the help of T cells. In an experiment, the susceptibility of mice treated with propionic acid to ventricular arrhythmias was significantly reduced, and the lesion area of aortic atherosclerosis was also significantly reduced (Bartolomaeus et al., 2019). Oleic Acid (OA) also plays a crucial role in the pathological process of cardiac remodeling. OA can significantly reduce the growth of cardiomyocytes and collagen expression of cardiac fibroblasts induced by angiotensin II (Ang II). OA supplementation can also prevent pathological cardiac remodeling by inhibiting the expression of fibroblast growth factor 23(FGF23) in the heart (Liu et al., 2020). Linoleic acid (LA; 18:2, n-6) and α- Linolenic acid (ALA; 18:3, n-3) inhibits the production of reactive oxygen species and downregulates the activation and transformation of growth factors of p38 MAPK pathway β 1 to play a protective role, transforming growth factor β. It plays a regulatory role in atrial fibrosis and promotes the progress of AF (Jiang et al., 2017). TMAO is produced by GM during the metabolism of fatty choline, and is also considered as a risk factor for cardiovascular disease. Li et al. concluded from the study on TMAO inducing myocardial hypertrophy and the expression of hypertrophy markers in Sprague Dawley rats *in vivo* and *in vitro* in neonatal ventricular myocytes that TMAO promotes apoptosis of vascular endothelial cells by inducing ROS through up regulation of the succinate dehydrogenase B (SDHB), thereby promoting the progress of atherosclerosis (Li et al., 2019). IS can promote atrial fibrosis and AF. IS exaggerates cardiac fibrosis in renal dysfunction by increasing reactive oxygen species through nicotinamide adenine dinucleotide phosphate (NADPH) oxidase. At the same time, IS has also been proved to increase oxidative stress and induce the production of reactive oxygen species by activating NADPH oxidase. It can be concluded that the activation of NADPH oxidase is related to the occurrence of AF (Aoki et al., 2015). LPS can induce systemic inflammatory response and increase the inducibility of AF. In the experiment, LPS treatment increased the expression of Cx43 protein, which plays a crucial role in the normal function of the cardiovascular system and is an important factor in the generation of arrhythmia (Chen et al., 2017). neonatal rat ventricular myocytes (NRVMs). activated by natural bovine deoxycholic acid or CDCA significantly induced FXR mRNA expression, while FXR mRNA was expressed in cardiomyocytes and rat cardiac fibroblasts. Activation of FXR could induce apoptosis and death of cardiomyocytes at early and late stages (Pu et al., 2013). In conclusion, there is ample evidence that GM and its metabolites can affect myocardial hypertrophy and fibrosis in a variety of ways, which most likely underlie the development of cardiac arrhythmias.

#### 4.1.4 Cardiomyocyte death

Structural remodeling is a characteristic pathological feature of many cardiovascular diseases, including AF and HF, which occurs through changes in cell proliferation, cell growth, cell apoptosis and vascular cell differentiation consistent with the homeostasis of blood vessels. CDCA has been proved to cause apoptosis of atrial cardiomyocytes, which may contribute to the evolution of structural remodeling. The elevation of serum CDCA plays a key role in the structural remodeling of AF in the metabolic patterns of non-RAF and RAF patients. CDCA is positively correlated with low voltage area of left atrium and might promote apoptosis of atrial myocytes (Wang et al., 2020). Butyric acid promotes the growth of vascular smooth muscle cells by inhibiting proliferation and apoptosis (Mathew et al., 2019). TMAO promotes vascular endothelial cell pyroptosis *via* ROS induced through succinate dehydrogenase complex subunit B upregulation, which may contributeto the progression of atherosclerotic lesions (Wu et al., 2020).

### 4.2 Electrophysiological remodeling

The heart functions like a mechanical pump, ensuring a continuous supply of blood to the system and the lungs. As a result, the human heart performs 100,000 successful and coordinated contractions per day. Due to the tight electrical regulation of the heart’s contractions, imbalances can cause problems with the rhythm of the heart. These contractions are controlled by electrical signals known as action potentials. Myocardial remodeling caused by GM imbalance is also reflected in electrophysiological remodeling (change of ion channel on the surface of myocardial cell membrane), slow inactivation of fast Na, reduced density of IKs, Ito and ICa-L, resulting in reduced cell coupling, slow conduction speed of action potential, shortened effective refractory period of myocardium, and promotion of reentry. For example, TMAO, a metabolite derived from gut microorganisms, aggravates the acute electrical remodeling in AF model by intensifying autonomic nerve remodeling (Yu et al., 2018). Additionally, it has been demonstrated that immune cells in persons who have dysbiosis of the GM differ from those in healthy people. Immune cells’ phenotypic and functionally different ion channels are influenced by how they are expressed. It is well known that leukocytes are abundant in the healthy myocardium, and diseases that produce arrhythmias, such as acute myocardial infarction, sepsis, heart failure, and myocarditis, alter their numbers, phenotypes, and electrophysiological characteristics. The most numerous leukocytes in the heart are macrophages, which are electrically connected to cardiomyocytes through gap junctions that contain gap junction protein43. This depot-derived connection causes rhythmic depolarization of macrophages and controls the resting membrane potential and action potential of cardiomyocytes. If macrophages are altered, this will greatly increase the risk of arrhythmias.

#### 4.2.1 Na ion channel

As is known to all, sodium channels are key channels for action potential generation, and the resulting changes in intracellular and extracellular sodium ion concentrations are important for cardiac action potential generation.GM is involved in the metabolism of BAs, and a possible mechanism for BA-induced arrhythmogenesis is the interaction with cell membranes and cell membrane ion channels or transporters. In an experimental study, Peter et al. discovered that taurocholic acid (TCA) induces changes in membrane potential through stimulation of the sodium-calcium exchange (NCX) in the myocardium, leading to increased NCX inward current density and resting membrane potential depolarization, which in turn affects myocardial electrical activity. Depolarization of the resting membrane potential and arrhythmias brought on by TCA are both prevented by NCX inhibition. However, it was also found that UDCA was ineffective in inducing arrhythmias, that UDCA coupling protected the cholesterol-rich plasma membrane from the toxic effects of hydrophobic BAs, and that UDCA caused hyperpolarization, thereby protecting cells from arrhythmias. Interestingly, AF patients had significantly lower serum levels of UDCA conjugates and higher levels of non-ursodeoxycholic acid (eg.TCA). This suggests that higher levels of toxicity (arrhythmogenic) and low levels of protective BAs create an environment of lower arrhythmia thresholds and therefore may promote arrhythmic events (Rainer et al., 2013).

#### 4.2.2 K ion channel

Cardiac action potentials are caused by complex but precisely regulated movements of ions across the myocardial membrane. Potassium channels represent the most diverse class of ion channels in the heart, where activation of the cardiac acetylcholine activated inward rectifier potassium current (IKACh) channels is an important component of the physiological control of cardiac function by the parasympathetic nervous system. In cardiac myocytes, KACh channels are coupled to muscarinic type 2 receptors (M2Rs) *via* Gi/o proteins, and when ACh binds to M2Rs, the βγ subunit of Gi/o proteins directly activates KACh channels, increasing its opening probability, thereby decreasing cardiac excitability. At present, a growing body of research has confirmed that constitutively activated IKACh is considered to be the background inward rectifier in persistent AF (PsAF) and therefore contributes to shortening the action potential duration and stabilizing the formation of high-frequency electrical rotors, which leads to PsAF (Noujaim et al., 2007). Ricardo A. et al. found that in canine and guinea pig atrial myocytes, cholinergic activation of IKACh, in a voltage-dependent manner, shortens the effective inactivity period and plays a role in the pathophysiology of AF (Navarro-Polanco et al., 2013). And furthermore, Sheikh et al. (2010) discovered that taurine-bound Bas also triggers potassium currents controlled by acetylcholine at muscarinic M2 receptors in cardiomyocytes, which may worsen AF. However, there is no direct evidence that alterations in GM can lead to arrhythmias due to abnormal ion channels pathological processes as described above, and these pathophysiology is often mediated through metabolites of GM, so we hypothesize that GM could affect IKACh channels and thus contribute to the development of arrhythmias.

#### 4.2.3 Ca ion channel

Arrhythmias are a complex group of diseases with many contributing factors, but changes in intracellular Ca^2+^ handling in cardiac myocytes are implicated in the pathogenesis of these diseases. Ca^2+^ is a pervasive second messenger that regulates a wide range of biological processes, including hormone production, muscular contraction, synaptic transmission, proliferation, and death. There is mounting evidence that AF and other heart disorders, including cardiac Ca^2+^ abnormalities, share this pathogenic pathway. Consequently, any impact on the cardiac calcium channels could result in arrhythmias. Indophenol sulfate is a protein-binding uremic toxin that builds up in CKD patients, and alterations in microbial fractions can cause an overproduction of uremic toxins. In an experimental study, Chen et al. found that indophenol sulfate increased the occurrence of pulmonary venous (PVs) and left atrial arrhythmias and decreased sinoatrial node pacemaker activity by causing oxidative stress and dysregulation of calcium handling in cardiomyocytes. This might be connected to how AF manifests in CKD patients (Chen et al., 2015). And given the evidence of simple Ca^2+^-driven localized activity and PV cardiomyocyte re-entry, PVs naturally play a crucial role in paroxysmal atrial fibrillation(pAF) patients. LPS down-regulates the expression of L-type calcium channels (a1C and b2 subunits) and abbreviates the effective refractory period (Okazaki et al., 2009). LPS can also upregulate the NLRP3 system, which in addition to causing structural remodeling can lead to re-entry of atrial action potentials promoting increased frequency of spontaneous sarcoplasmic reticulum Ca^2+^ release during systole and diastole, which may lead to delayed post-depolarization and trigger ectopic activity, thereby affecting myocardial electrical activity (Heijman et al., 2020; Liu et al., 2021). These mechanisms may provide pieces of evidence for Ca regulation to promote arrhythmia.

### 4.3 Nervous system regulation

The gut-brain axis’s bottom-up signaling is poorly understood. The activation of immune cells in the stomach, which subsequently go to the brain, is the pathway that has received the most research. Benakis et al. (2016) showed proof that the health of the GM affects the polarization of naive T lymphocytes in the gut. After proximal middle cerebral artery occlusion (MCAO), animals with an anti-inflammatory microbiome performed better than mice with a pro-inflammatory microbiome.

However, the brain-heart axis’s up-bottom signaling has been investigated extensively. Through both autonomic nervous and vagus nerve system and system, the nervous system may control heart rate. Arrhythmias can arise as a result of the central nervous system’s ability to restrict parasympathetic tone while raising sympathetic tone. The mechanism by which the heart is regulated by the nervous system is very complex. There might be hundreds to thousands of autonomic neurons in the implanted ganglion plexus (GP). At least seven large GPs may be seen in the human heart, including four big left atrial GPs that are situated close to the pulmonary veins. Autonomic ganglia are also identified inside the Marshall’s ligament based on clinical anatomy, particularly their placement to certain PVs. These GPs serve as integration hubs, connecting the cardiac autonomic nervous system (ANS) with autonomic innervation. Despite the GPs having four parasympathetic and four sympathetic components, the parasympathetic component predominates. Notably, several sympathetic and vagus nerve branches go straight to the heart instead of going through the GP (Kawashima, 2005).

#### 4.3.1 Autonomic nervous system

The formation and maintenance of cardiac arrhythmias depend heavily on the autonomic nervous system. Extrinsic and intrinsic components of the cardiac ANS can be arbitrarily separated. Neurons in the brain and spinal cord as well as nerves connecting to the heart make up the extrinsic cardiac ANS. In the heart itself and along the large veins of the thorax, autonomic neurons and nerves make up the majority of the intrinsic cardiac ANS. In canine models, local injection of TMAO activates the atrial autonomic ganglion plexus and promotes arrhythmia, possibly by activating p65 nuclear factor-κB signaling and increasing expression of inflammatory cytokines (Yu et al., 2018). It has been demonstrated that the pathophysiology of AF or ventricular arrhythmia(VA) is modulated by the cardiac autonomic nerve system (CANS) (Shivkumar et al., 2016).

There is proof that the left cardiac sympathetic nerve is hyperactive before VA develops, and activation of the left stellate ganglion (LSG) can greatly increase the likelihood of VA (Yu et al., 2017). Meng et al. (2019) directly activated LSG by local injection of TMAO into LSG and indirectly activated LSG by systemic injection of TMAO to stimulate the central sympathetic nervous system. The findings demonstrated that intravenous and local TMAO treatment significantly increased LSG function and activity in comparison to the control group, which elevated cardiac sympathetic tension, decreased the effective refractory period, and worsened ischemia-induced VA. At the same time, TMAO can significantly promote the expression of proinflammatory markers, such as IL-1, IL-6, TNF-α, and N-Methyl-d-Aspartate Receptor (NMDAR), which may further cause an overactive sympathetic nervous system in LSG and encourage the development of VA.

#### 4.3.2 Vagus nerve system

Regulating cardiac rhythm is negatively impacted by the vagus nerve. The major cardiac branch of the vagus trunk is where the recurrent laryngeal nerve is connected. According to functional studies, the majority of the vagal fibers congregate in a unique fat pad (referred to as the third fat pad) between the superior vena cava and the aorta on their journey to the sinoatrial and atrioventricular nodes (Chiou et al., 1997). Vagal discharge enhances IKACh, reduces action potential duration (APD) and stabilizes the folding rotor (Kneller et al., 2002), while adrenergic receptor activation increases L-type calcium current (ICa-L), ryanodine receptor opening probability and SR Ca^2+^ load through calmodulin-dependent protein kinase II and protein kinase phosphorylation (Dobrev et al., 2011).

Microbiota elements can directly or indirectly trigger vagus nerve(VN) afferent fibers through the gut endocrine cells (GEC). Through the central autonomic network, VN afferent fibers stimulate the central nervous system (CAN). Through inflammatory reflexes, VN afferent fibers can trigger efferent fibers. By improving tight junctions, VN efferent fibers can decrease gastrointestinal tract inflammation and gut permeability (Bonaz et al., 2018). Depending on the substance, the different SCFAs produced by microbiota maybe activate vagal afferent fibers in different ways. For example, oleic acid, a long fatty acid, acts on vagal afferent fibers *via* a cholecystokinin (CCK)-mediated mechanism, whereas Butyric Acid, a short fatty acid, directly affects afferent terminals (Lal et al., 2001).

### 4.4 Other diseases related to arrythmia

#### 4.4.1 Hypertension

GM can affect blood pressure, and hypertensive patients are involved in arrhythmias through multiple modes of action. The relationship between hypertension and cardiac arrhythmias is complex (Nattel, 2002). The incidence of AF, VA, and sudden death were positively correlated with the course of hypertensive heart disease as manifested by cardiac remodeling. Mechanisms and manifestations of arrhythmogenesis in hypertensive patients Hypertension affects the development of arrhythmias through macroscopic and microscopic changes in the cardiac environment, leading to electrical and structural alterations. hypertension further promotes left ventricular hypertrophy (LVH), which is a risk factor for arrhythmias. Chronically elevated afterload and intracardiac pressure encourage cardiomyocyte hypertrophy and activate myocardial fibroblasts. Enhanced myocardial mass as a result of increased cardiomyocyte hypertrophy and collagen deposition from myocardial fibroblasts causes LVH (Kahan and Bergfeldt, 2005). It has been established that LVH increases the risk of developing arrhythmias, possibly by inducing myocardial ischemia and an increase in myocardial oxygen demand as well as LV diastolic dysfunction. Myocardial scarring, systolic LV dysfunction, and potentially harmful arrhythmias can all result from intermittent myocardial ischemia with temporary diastolic failure. Importantly, the risk of AF is also increased. And Juan et al. also demonstrated that diastolic dysfunction is an initial functional maladaptation of LVH with progressive remodeling leading to decreased systolic function (Figueroa et al., 2010). Blood pressure was taught to be lower in germ-free mice deficient in GM than in conventional mice. Meanwhile, transplantation of feces from human hypertensive donors into germ-free mice resulted in higher blood pressure in these mice. A reduction in the number of bacterial species with diastolic metabolite producing properties in the hypertension model also suggests that GM affects blood pressure (Adnan et al., 2017). For example, butyric acid -producing bacteria and butyric acid levels are relatively low in hypertensive patients (Huart et al., 2019). However, the same metabolites may produce conflicting biological effects through different receptors. For example, Jennifer et al. found that propionic acid may upregulate blood pressure through olfactory receptor 78 (Olfr78), while also exerting a hypotensive effect through activation of Gpr41 (Pluznick et al., 2013).

#### 4.4.2 Atherosclerosis

Atherosclerosis is also one of the risk factors for inducing arrhythmia. When atherosclerosis affects the conduction system, impeding impulse conduction, it will cause various arrhythmias. Gut microbes are involved in atherosclerosis through their metabolites. Alterations in gut microbiome composition were confirmed in a genome-wide association study including 218 patients with atherosclerosis and 187 healthy controls. Specifically, patients with atherosclerosis had a significant increase in the abundance of *Escherichia coli*, *Klebsiella* spp., and Enterobacter aerogenes, and a decrease in the abundance of two butyric acid-producing bacteria, *Roseburia gutis* and *Faecalibacterium cf. prausnitzii* (Jie et al., 2017). Patients with coronary artery disease (CAD) have a lower number of Gram-negative gut microorganisms compared to controls (Yoshida et al., 2018). Metabolites of gut microbes such as IS and TMAO can be involved in atherosclerosis by increasing the production of endothelial reactive oxygen species and impairing endothelial-mediated vasodilation. It has been shown that direct exposure to TMAO can further enhance platelet activation *via* a number of agonists, a process mediated by enhanced release of intracellular Ca^2+^ stores (Nam, 2019). Indodione sulfate has been shown to negatively affect the protective properties of endothelial cells (e.g., migration and duct formation) by depleting the bioavailability of nitric oxide at higher concentrations (Kharait et al., 2011). IS affects cellular senescence and, together with asymmetric dimethylarginine, exacerbates aortic calcification in hypertensive rats (Adijiang et al., 2010). Meanwhile, the mechanisms by which gut bacteria are involved in atherosclerosis have guided new directions in their treatment of atherosclerosis. Kasahara and his colleagues demonstrated that *Roseburia* sp. can ameliorate atherosclerosis by shaping gene expression, enhancing fatty acid metabolism, and reducing the inflammatory response (Kasahara et al., 2018). Oral gavage of butyric acid altered microbiota composition and enhanced gut microbial diversity. butyric acid treatment significantly reduced atherosclerotic plaque formation through upregulation of ABCA1 and subsequent cholesterol efflux (Du et al., 2020). CAD due to gut microbiota-induced atherosclerosis may provide a susceptibility mechanism for the development of arrhythmias.

Acute myocardial infarction (AMI)is a serious consequence of atherosclerosis, which frequently occurs in conjunction with VA, particularly VT and VF, which can raise the risk of sudden mortality and a poor prognosis for patients while they are being treated in the hospital.VA following acute myocardial infarction is significantly correlated with a number of inflammatory markers and electrophysiological alterations. In mice with myocardial infarction, Bacteroides fragilis raised the number of Foxp3+Treg cells, promoted the release of anti-inflammatory cytokines, and reduced ventricular remodeling (Severs et al., 2008). Changes in cardiac gap junction proteins, including the decrease of Cx43 and spatial redistribution, are characterized by abnormal electrophysiological remodeling and are referred to as representing arrhythmogenic substrates (Severs et al., 2008). Zhou et al., (2021) found that propionic acid can improve the pathological remodeling of connexin in rats with myocardial infarction. propionic acid causes the nucleus tractus solitarius in the brain through the nodular ganglia to activate vagal afferent fibers in the gut and vagal efferent neurons that innervate the heart (Dalile et al., 2019). Increased nerve excitability and inflammatory response are closely associated (Wernli et al., 2009; Wang et al., 2017). Proinflammatory cytokines decrease VA following myocardial infarction by causing gap junction remodeling, reduced expression, and/or redistribution of Cx43 (George et al., 2017).

#### 4.4.3 Type 2 diabetes and obesity

Early work in the field of GM shows that the imbalance of normal microbiota may lead to many inflammatory diseases, among which obesity and insulin resistance are the main diseases caused by the imbalance of GM and energy imbalance. At the same time, abnormal glucose tolerance and obesity are one of the important causes of arrhythmias. The incidence of atherosclerosis and the probability of thrombosis will increase in patients with diabetes, which will lead to acute coronary artery ischemic heart disease or arrhythmia.

Type 2 diabetes is associated with moderate and severe gut disorders. Data from metagenomic analysis shows that in the gut microbial imbalance of type 2 diabetes patients, the abundance of some common butyric acid producing bacteria decreases, various conditional pathogens increase, other microbial functions are abundant, and sulfate reduction and oxidative stress resistance are enhanced (Qin et al., 2012). Another study also found that the improvement of insulin response after oral glucose tolerance test was related to the increase of butyric acid driven by the host gene, while the increase of Type 2 diabetes risk was related to the abnormal production or absorption of propionic acid (Sanna et al., 2019). The GM of obese animals and human subjects was significantly different from that of thinner subjects. Recently, the overall reduction of bacterial diversity and the change of bacterial gene expression are considered to be the main reasons affecting metabolic pathways, which may be related to obesity. Adipose tissue is the main target of metabolites produced by GM. One of the important functions of gut microorganisms is to decompose the substrate into SCFAs. In the interaction between short chain fatty acids and the host, the role of GPCRs located in adipose tissue cannot be ignored. Microorganism transplantation can spread the increased fat in the host. Later, it was found that the destruction of the microbiota in early life would lead to increased obesity (Mishra et al., 2016). Arrhythmia is a process that occurs slowly and has complex mechanisms. From the perspective of intestinal flora and its metabolites, clinicians can gain a new perspective on preventing and treating diabetes/obesity combined with arrhythmia. Still, a large number of experiments need to be confirmed.

#### 4.4.4 NAFLD

Non-alcoholic fatty liver disease (NAFLD) is a clinicopathological syndrome characterized primarily by excessive intracellular fat deposition in the liver, excluding alcohol and other definite liver-damaging factors, and is the most common liver disease. There is accumulating evidence that NAFLD is a multisystem illness that may lead to IR, poor lipid and glucose metabolism, inflammation, and oxidative stress, among other systemic deleterious consequences (Targher et al., 2020). These alterations may act synergistically to enhance structural, electrical, and autonomic remodeling of the heart, hence raising arrhythmia susceptibility. In addition, pericardial fat volume or thickness correlates with the prevalence and severity of AF, with most reports showing a positive correlation between NAFLD and epicardial fat volume or thickness, while Ze Chen et al. found significantly higher EAT in patients with NAFLD compared with controls through their study, which correlated with the severity of steatosis (Chen et al., 2021), which may also contribute to the arrhythmias induced by NAFLD. Furthermore, the gut-liver axis is the link between the gut microbiota and the liver. Gut flora dysfunction could lead to PAMP (pathogen-associated molecular patterns) production, increased mucosal barrier permeability leading to liver inflammation and the development and progression of liver disease, and it has been found that microbiota diversity is lower in patients with NAFLD compared to healthy controls (Drozdz et al., 2021). Moreover, cardiac arrhythmias are significantly impacted by intestinal problems in a variety of ways.

In conclusion, NAFLD is closely related to arrhythmias, but the available evidence is not perfect and requires our continued research.

#### 4.4.5 OSAHS

Obstructive sleep apnea hypoventilation syndrome (OSAHS) is a condition that causes apnea and hypoventilation while you sleep and is characterized by snoring, a disrupted sleep pattern, frequent drops in oxygen saturation, and daytime tiredness. Patients with OSAHS have been linked to the development of AF through a number of pathophysiological pathways, including apnea-induced hypoxia, intrathoracic pressure shift, sympathetic imbalance, atrial remodeling, oxidative stress, inflammation, and neurohumoral activation. Particularly in OSAHS patients, hypoxia during sleep promotes sympathetic activity, which is a critical mechanism for inducing AF. Therefore, it is possible that OSAHS itself contributes to the development of AF (Goudis and Ketikoglou, 2017). Studies are now showing that people with AF have an increase in the gut type dominated by *Ruminococcus gnavus*, similar findings were made by Ko, C.Y. et al. in a study of OSAHS (Ko et al., 2019). We therefore hypothesize that the common cause of the pathophysiological processes of OSAHS and AF may be due to similar alterations in GM, especially an increase in *Ruminococcus gnavus*, which needs further research.

#### 4.4.6 COVID-19

The SARS-CoV-2 virus primarily affects the upper respiratory tract, but it can cause life-threatening lung problems. The GM Is extensively disrupted in some people with mild to severe COVID-19, and this disruption can remain for up to one month (Deidda et al., 2021). As with other respiratory viral infections, COVID-19 can be associated with gastrointestinal symptoms such as nausea, vomiting, abdominal pain, and diarrhea. Because the pathways affecting the gut tract exist through the immune and nervous systems, which not only respond to GM but also regulate its composition, the disturbance of GM caused by respiratory infections does not necessarily depend on the active replication of the virus in the intestine. The same embryonic foregut gives rise to both the intestine and the lungs during development. Both organs serve as a mucosal barrier between the deep tissues and the outside world after birth. Circulating lymphocytes, a direct immunological contact between the two organs since these cells do not stay in one place, patrol both the intestine and airway mucosa. The “gut-lung axis” is the crosstalk in which these organs “communicate.” In conclusion, GM triggers arrhythmias through various secretions, and the SARS-CoV-2 virus may be a risk factor for arrhythmias due to the presence of the “entero-pulmonary axis”.

## 5 Treatment

### 5.1 Components of the diet: Eat more beneficial lipids and small molecules of dietary fiber

Some studies have investigated the effects of different food components and dietary patterns on gut flora, which could be an important target for future treatment of arrhythmia through GM. A recent study showed that AF patients favored to take more energy from animal fat (Tabata et al., 2021). As for the correlation between GM and fat intake, the phylum Bacteroidetes showed a weak negative correlation with fat intake (notably animal fat) in AF patients, whereas Firmicutes were found the opposite correlation. Metabolic endotoxemia and chronic inflammation may be the mechanism by which fat intake induce AF. Intake of OA significantly contributes to a reduced risk of cardiovascular disease since it could inhibit oxidative stress to lessen cardiomyocyte damage (Singh et al., 2020). The Mediterranean diet can beneficially affect the GM and related metabolome (De Filippis et al., 2016). The vegetable-based diet population has a better microbial metabolome. A study in the AF patients showed that Mediterranean diet may lower oxidative stress to reduce risk of AF (Pastori et al., 2015).

### 5.2 Probiotics and prebiotics

#### 5.2.1 Probiotics

The current definition, formulated in 2013 by the experts of the International Scientific Association for Probiotics and Prebiotics (ISAPP), states that probiotics are “live microorganisms that, when administered in adequate amounts, confer a health benefit on the host” (Hill et al., 2014). “Live strains of strictly selected microorganisms which, when administered in adequate amounts, confer a health benefit on the host”. Hill et al. (2014) proposed four mechanisms for the beneficial effects of probiotics on human health: (1) enhancement of barrier function; (2) competition with pathogens by using nutrients and adhesion sites; (3) the impact of neurotransmitter production and immune system on other organs; (4) immunomodulation. The current definition, formulated in 2002 by FAO (Food and Agriculture Organization of the United Nations) and WHO (World Health Organization) working group experts, states that probiotics are “live strains of strictly selected microorganisms which, when administered in adequate amounts, confer a health benefit on the host”. Several clinical studies summarized by Borja Sánchez et al. demonstrated the positive effects of probiotics in the treatment of gastrointestinal disorders (e.g. diarrhea, Inflammatory bowel disease, irritable bowel syndrome, Helicobacter pylori infection, gastrointestinal disorders, inflammatory bowel disease).In another clinical trial, carried out among diabetic people with CHD, the researchers conclude that vitamin D plus probiotic might reduce inflammation of body and improve antioxidant capacity, NO, glycemic control and HDL level (Raygan et al., 2018). Chen et al. found oral administration of L. acidophilus ATCC 4356 can alleviate the progression of atherosclerosis by modulating oxidative stress and inflammatory processes in ApoE-/- mice (Chen et al., 2013). Probiotics can also affect the metabolism of drugs. Amiodarone, the principal antiarrhythmic drug on the market, has extracardiac toxicity, so researchers focus on finding ways to improve the metabolism of residual amiodarone in the body. Banoth et al. (2020) found Saccharomyces cerevisiae strain OBS2 which is a therapeutic potential probiotic could enhance degradation of residual amiodarone *in vitro* and *in vivo*.

#### 5.2.2 Prebiotic

Prebiotic is defined as “a substrate that is selectively utilized by host microorganisms conferring a health benefit” by ISAPP in 2016 (Gibson et al., 2017). A prebiotic is a substance from which an organism obtains its nourishment, besides its health benefit depends on microbial metabolism. Therefore, prebiotics are considered as a substitute or supplement for probiotics and stimulate the growth and activity of beneficial bacteria in the gastrointestinal tract (GIT). The current dominance of Fructans (fructooligosaccharides (FOS) and inulin) and galactans (galactooligosaccharides or GOS) in the prebiotic category has been confirmed by dozens of studies (Gibson et al., 2017). Inulin, FOS and GOS are considered safe food ingredients so they can be added to food to increase nutrition and health value. The growing evidence summarized by Corrie M Whisner (Whisner and Castillo, 2018) showed that prebiotic effects increase calcium absorption and improve bone health. In addition, prebiotics can increase abundance of *bifidobacteria* which is one of the probiotics in the GIT to prevent gut inflammation (Simpson and Campbell, 2015). Another study conducting in the rat model of ischemia-reperfusion concluded Larch arabinogalactan could Inhibit apoptosis to reduce myocardial injury (Lim, 2017).

### 5.3 Fecal microbiota transplantation

Fecal microbiota transplantation as a safe therapeutic method is widely used in gastrointestinal and extra-gastrointestinal diseases (Wang X, et al., 2019). Spychala et al. (2018) found that *Faecalibacterium prausnitzii* treatment improved liver health and reduced inflammation in adipose tissue in mice, which could be a potential treatment for arrhythmia with GM. Recently, Kong et al. (2022) transplanted fecal microbes derived from high-fat diet (HD)-fed mice to normal diet (ND)-fed mice and they found the susceptibility to AF increased significantly. ND-fed mice receiving FMT from mice on a high-fat diet suffered metabolic endotoxemia caused by *Desulfovibrionaceae* which highly increased, in addition to elevated circulating LPS, disrupted gut tissue structure, and increased left atrial proinflammatory factors.

### 5.4 Interaction of medication and GM

Normal GM can effectively resist infection by pathogenic bacteria, but the use of drugs can affect the composition of GM. Therefore, we should take into account the effects of the drugs on the GM. For example, proton pump inhibitor (PPI) is routinely used in patients with AF undergoing catheter ablation to prevent left atrial esophageal fistula. However, oral PPI will change GM (Imhann et al., 2016), increase the risk of gut infection and reduce the health of GM. The decreased concentration of gastric acid is due to the use of PPI, leading to the weakened barrier function and increased number of the order *Actinomycetales*, families *Micrococcoceae*, *Streptococcoceae*, genus *Rothia* and species *Lactobacillus salivarius*. At present, it is not clear whether the abundance of *streptococcus* and *parabacitracin* in patients with AF is affected by PPI, and further research is needed to clarify. Patients with cardiac arrhythmias are often found with atherosclerosis and need antiplatelet drugs, such as aspirin, to be taken. A study in the mice model of colorectal cancer receiving aspirin demonstrated an increase in the beneficial bacteria Bifidobacterium and Lactobacillus genera and a reduction in the pathogenic bacteria of the GM. And Further research is needed to determine the effect of GM in patients with arrhythmias taking aspirin (Zhao et al., 2020). Diabetes is also a common co-morbidity in people with arrhythmias nowadays. According to Vallianou et al. summarizing studies of gut microbiology in patients with type 2 diabetes, a decrease in butyrate-producing bacteria was found, from which it was inferred that the use of the hypoglycemic agent metformin may also affect changes in the gastrointestinal microbiome (Vallianou et al., 2019). A recent study found that metformin may improve liver injury by adjusting GM and lessening colonic barrier dysfunction in septic rats, which may be an effective treatment for sepsis-related liver injury (SLI) in the future (Liang et al., 2022). Anticoagulation is an important part of the treatment strategy for patients with AF. In a model of GM dysbiosis constructed using amoxicillin, GM diversity was reduced after amoxicillin treatment. In addition, warfarin and rivaroxaban bioavailability was significantly higher in the amoxicillin-treated group, whereas dabigatran bioavailability increased in the 3-day amoxicillin-treated group but significantly decreased in the 7-day amoxicillin-treated group (Chen et al., 2022).

### 5.5 Immunomodulator

Histone deacetylases (HDACs) play vital roles in calcium homeostasis, AF genesis and heart failure, so HDAC inhibitors might be are the potential treatment. Lkhagva et al. (2018) concluded that HDACi (MPT0E014, MS-275) by rescuing mitochondrial bioenergetics to therapy cardiac dysfunction which is TNF-α induced mitochondrial dysfunction with lowering energy availability. Another research conducted in rabbits with AF shows that HDACi could decrease calcium homeostasis induced AF and pulmonary vein(PV) arrhythmogenesis in which rabbits had less AF and shorter AF duration. The decrease of calcium transient amplitude, sodium-calcium exchange current and ryanodine receptor expression in PV cardiomyocytes treated with MPT0E014 might be the potential mechanism of HDACi in the treatment of AF (Lkhagva et al., 2014).

## 6 Conclusion

Evidence from numerous studies in both humans and animals suggests that the role of the GM and its metabolites in arrhythmia is well established. Through a variety of pathways, the GM significantly affects cardiac arrhythmia and provides a wide range of potential therapeutic approaches. Nevertheless, the exact mechanism of the cross talk between the gut microbes and the heart still needs to be investigated in depth, and key molecules, regarded as key therapeutic targets, need to be further explored.

## Author contributions

HF, BL and FZ designed structure of this study; XL performed the literature search. HF and XL completed the figures and tables; ZR, XF, JL, YX, XY and FZ edited and revised the manuscript. HF and XL drafted the manuscript. All authors contributed to the article and approved the submitted version.
